# Supplementary figures and images for: Neutrophil-to-lymphocyte ratio associated with an increased risk of mortality in patients with critical limb ischemia
Source: PLoS One. 2021 May 27;16(5):e0252030. doi: 10.1371/journal.pone.0252030 (PMC8158906; doi:10.1371/journal.pone.0252030)

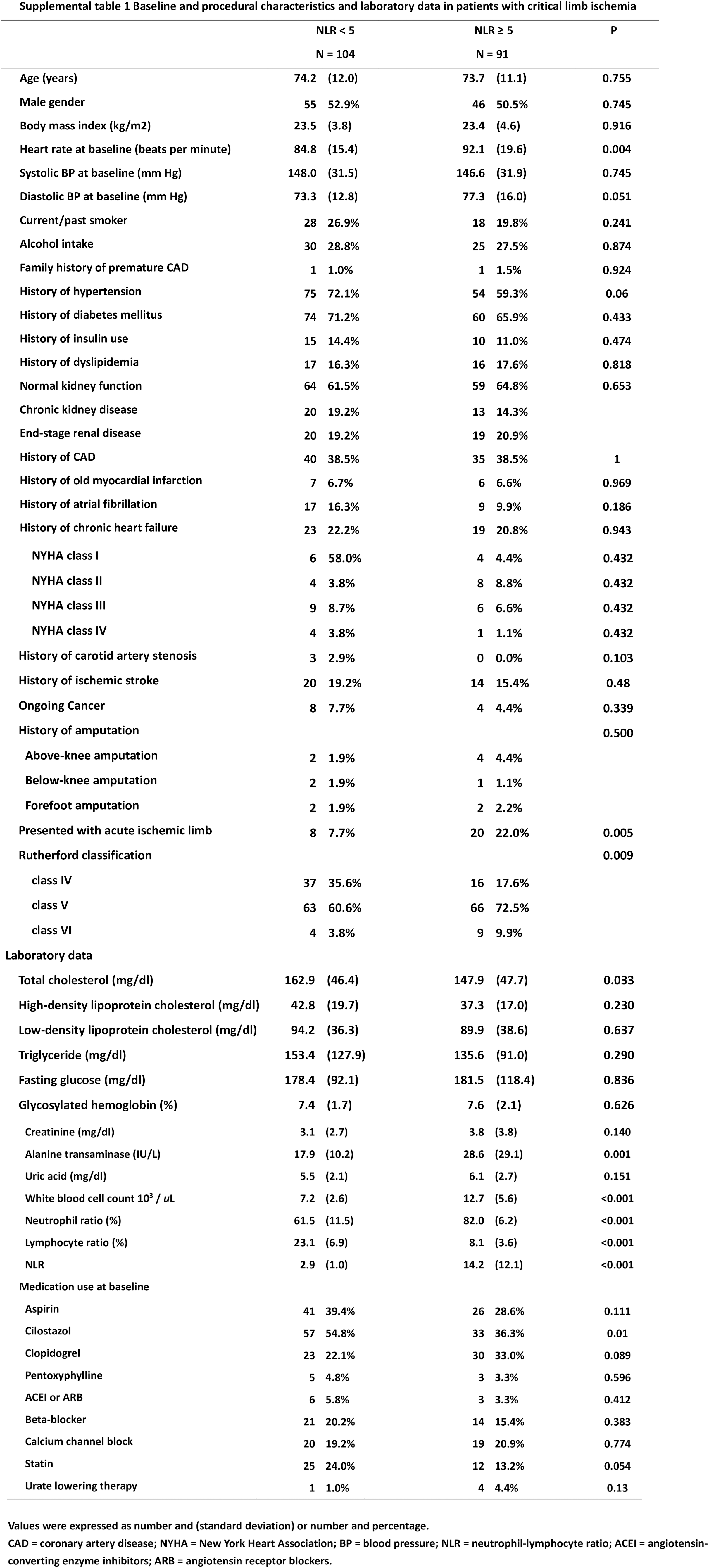

Supplement: S1 Table — Values were expressed as number and (standard deviation) or number and percentage. CAD = coronary artery disease; NYHA = New York Heart Association; BP = blood pressure; NLR = neutrophil-lymphocyte ratio; ACEI = angiotensin-converting enzyme inhibitors; ARB = angiotensin receptor blockers. (TIF) [file pone.0252030.s001.tif]

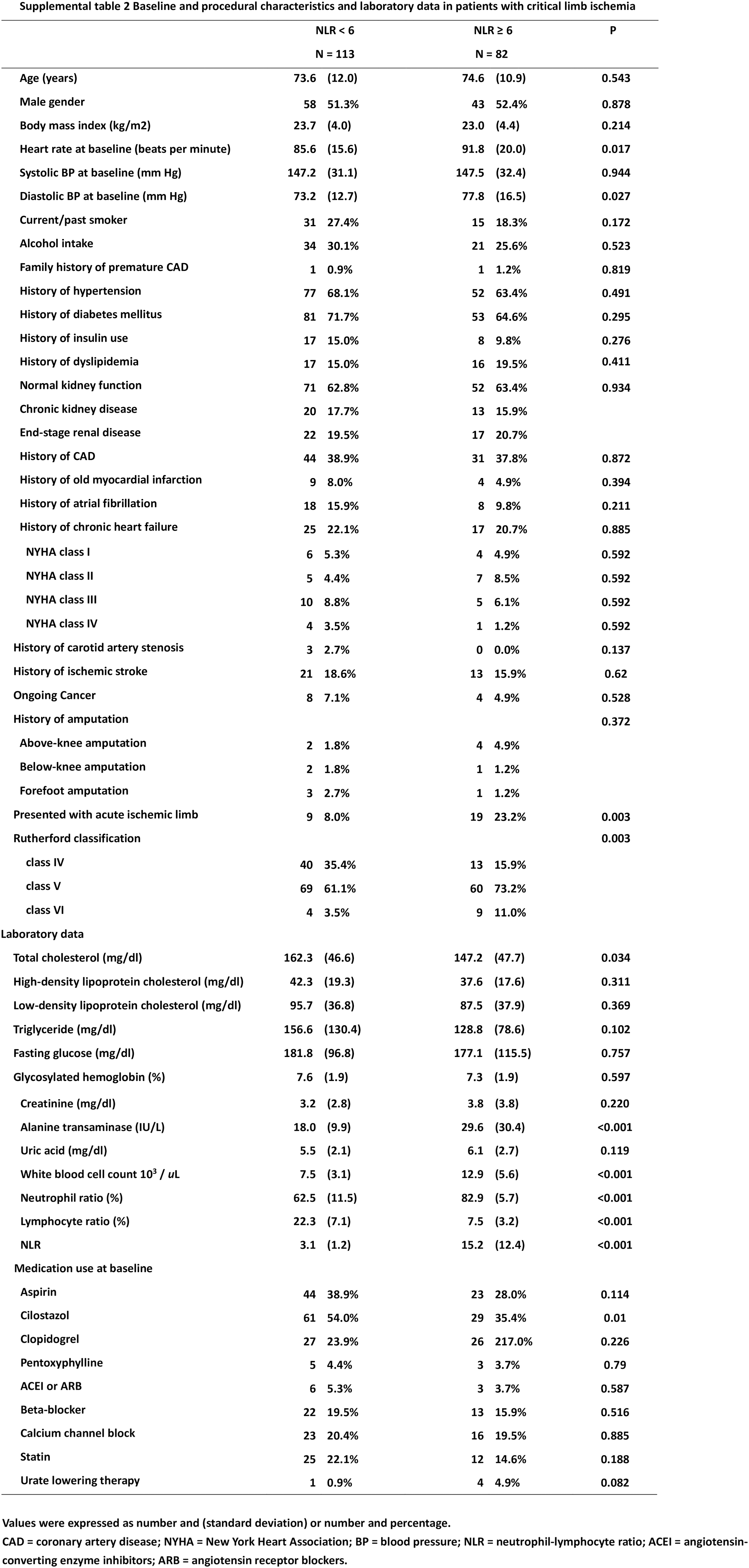

Supplement: S2 Table — Values were expressed as number and (standard deviation) or number and percentage. CAD = coronary artery disease; NYHA = New York Heart Association; BP = blood pressure; NLR = neutrophil-lymphocyte ratio; ACEI = angiotensin-converting enzyme inhibitors; ARB = angiotensin receptor blockers. (TIF) [file pone.0252030.s002.tif]
